# 3D osteotomies—improved accuracy with patient-specific instruments (PSI)

**DOI:** 10.1007/s00068-022-02060-4

**Published:** 2022-07-26

**Authors:** Maximilian Jörgens, Alexander M. Keppler, Philipp Ahrens, Wolf Christian Prall, Marcel Bergstraesser, Andreas T. Bachmeier, Christian Zeckey, Adrian Cavalcanti Kußmaul, Wolfgang Böcker, Julian Fürmetz

**Affiliations:** 1 Department of Orthopaedics and Trauma Surgery, Musculoskeletal University Center Munich (MUM), University Hospital, LMU Munich, Munich, Germany; 2 OrthoPlus Munich, Munich, Germany; 3 OT Medizintechnik GmbH (Medical Engineering in Orthopedics and Traumatology), Munich, Germany; 4 Department of Trauma Surgery and Orthopaedics, RoMed Klinikum Rosenheim, Rosenheim, Germany; 5 Department of Trauma Surgery, BG Unfallklinikum Murnau, Murnau, Germany; 6 FIFA Medical Centre of Excellence, Division of Knee, Hip, Shoulder and Ellbow Surgery, Schoen Clinic Munich, Munich, Germany

**Keywords:** 3D-planning, HTO, Open-wedge tibial osteotomy, Patient-specific instruments, PSI, Spacer

## Abstract

**Purpose:**

Three-dimensional (3D) printed patient-specific instruments (PSI) have been introduced to increase precision and simplify surgical procedures. Initial results in femoral and tibial osteotomies are promising, but validation studies on 3D planning, manufacturing of patient-specific cutting blocks and 3D evaluation of the attained results are lacking.

**Methods:**

In this study, patient-specific cutting blocks and spacers were designed, fabricated, and used to perform a high tibial osteotomy (HTO). After segmentation of CT data sets from 13 human tibiae, 3D digital planning of the HTO was performed with a medial opening of 8 mm. These 3D models were used to fabricate patient-specific cutting blocks and spacers. After the surgical procedure, accuracy was evaluated measuring 3D joint angles and surface deviations.

**Results:**

The lowest mean deviation was found to be 0.57° (SD ± 0.27) for the MPTA. Medial and lateral tibial slope deviated from the 3D planning by an average of 0.98° (SD ± 0.53) and 1.26° (SD ± 0.79), respectively, while tibial torsion deviated by an average of 5.74° (SD ± 3.24). Color analysis of surface deviations showed excellent and good agreement in 7 tibiae.

**Conclusion:**

With 3D cutting blocks and spacers, the 3D planning of the HTO can be translated into reality with small deviations of the resulting joint angles. Within this study, the results of the individual steps are examined for errors and thus a critical evaluation of this new and promising method for performing patient-specific HTOs is presented.

## Introduction

Medial open-wedge high tibial osteotomy (HTO) is an effective procedure to adjust the coronal and sagittal alignment of the tibial plateau [1–4]. The need for coronal alignment correction can be indicated by isolated medial tibiofemoral compartment osteoarthritis in younger and physically active patients. The correction can delay the progression of osteoarthritic joint degeneration with a probability of survival between 85.4 and 91.6% at 10 years [1, 5, 6]. On the other hand, sagittal tibial slope correction can be used as a powerful tool in patients with cruciate ligament deficiencies or prior to the ligament reconstruction to prevent mechanical failure of the graft [7–9].

Conventional HTO largely remains an unguided procedure involving preoperative two-dimensional (2D) planning based on standing full-leg radiographs [10]. In clinical practice, analysis and planning of deformity correction are performed by the treating surgeon using 2D radiographs, based on commonly known definitions and criteria (e.g., Paley Book) [11]. But three-dimensional (3D) changes in the anatomy cannot sufficiently be depicted or planned with these images and occur unintentionally in many cases of HTO [12–14]. However, 3D imaging is possible with computer tomography (CT) and magnetic resonance imaging (MRI), and 3D HTO planning, as well as surgical implementation with 3D-printed PSIs, shows promising clinical results [15–18]. However, the 3D analysis and planning procedures are complex and constitute intellectual property of the manufacturing companies. Although the physician is involved in the planning process, potential sources of error and validation of the individual steps often remain unknown. Therefore, the group developed a standardized, validated 3D analysis and implemented reference values based on 3D analysis of a young cohort [19–21].

To better evaluate the implementation of 3D HTO planning, 3D printed patient-specific instruments (PSIs) and spacers were designed, manufactured and used in this study. To avoid large soft tissue detachment, the 3D printed cutting blocks were designed to be smaller than in previous published studies [15, 16].

Based on the group`s investigations, it was hypothesized that an accurate tibial realignment can be performed with the help of 3D planning. 3D planning and postoperative results were compared by different means: Medial proximal tibial angle (MPTA), medial and lateral tibial slope as well as tibial torsion.

## Material and Methods

The study consisted of three steps. In the first step, 13 surface models of human tibiae created with CT scans were subjected to standardized 3D HTO planning using 3D software. Second, patient-specific sawing templates and spacers were created based on the virtual planning before the HTO (8 mm standardized gap) procedure took place. Third, the realigned tibiae were reevaluated by additional CT scan and the joint angles were compared with the original 3D plan. Furthermore, discrepancies between plans and outcomes were visualized by false color analysis.

### Generation of a surface model and standardized 3D planning of the HTO

**Fig. 1:**
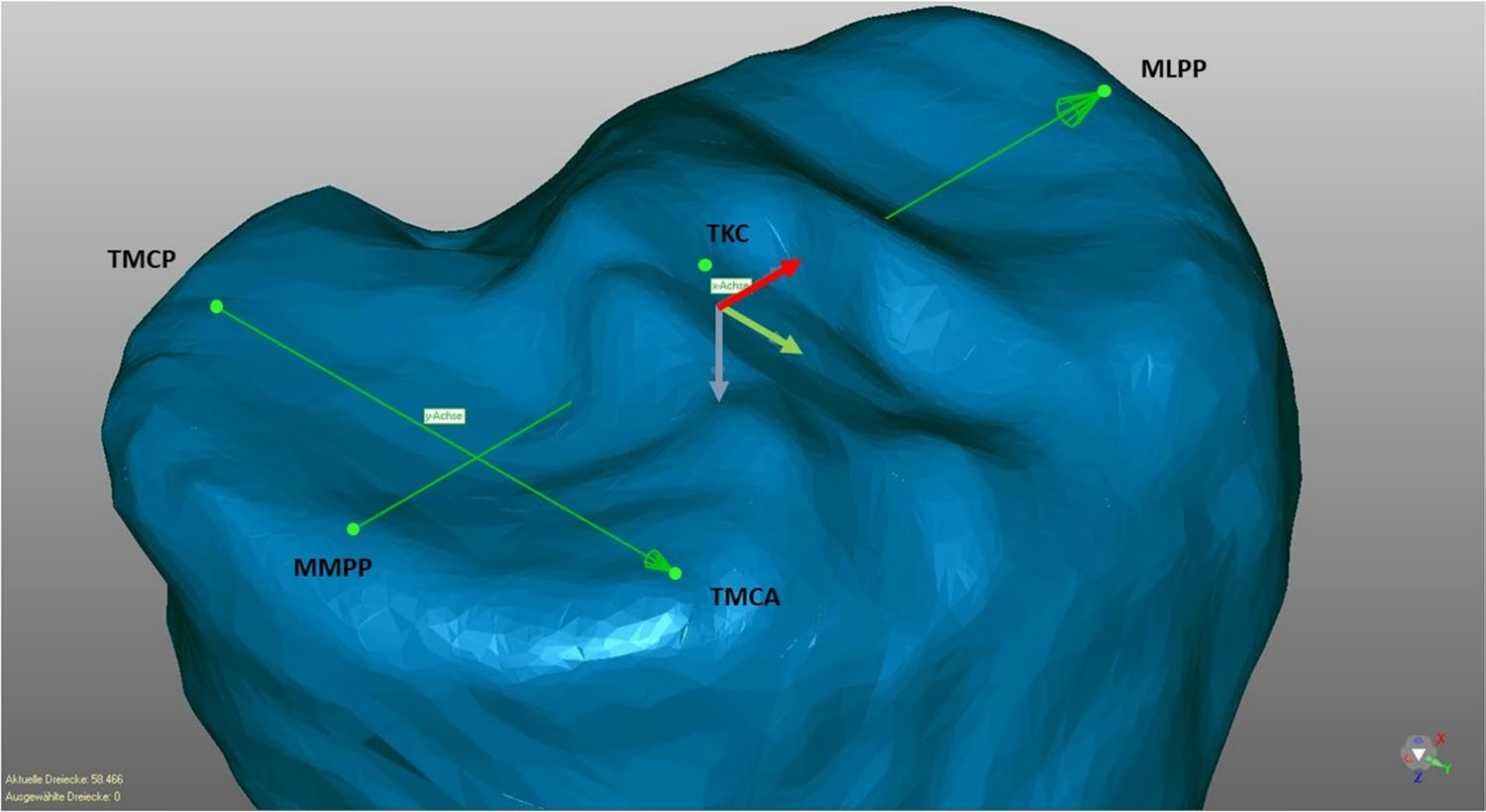
3D model in a coordinate system (x-axis = red; y-axis = green; z-axis = violet). y-axis is defined by the tibial most proximal medial anterior point (TMCA) and the tibial most proximal medial posterior (TMCP) of the medial plateau. The xy-plane is defined by y-axis and a parallel running to a line between most medial and lateral proximal points (MMPP, MLPP). z-axis runs perpendicular to xy-plane [through TKC (tibial knee center)]. TMCA, TMCP define the medial slope (view from anteromedial proximal)

The 13 human cadaveric fresh frozen tibiae without any soft tissues were scanned using CT (slice thickness 0.625 mm; GE HD750 CT, General Electric, Boston, USA). Based on the segmented DICOM data, a surface model (STL file) was generated based on a previously published method by semi-automatic watershed segmentation of the CT data sets utilizing the ImFusion Suite [22]. The resulting surface model was then transferred to the 3D software Geomagic Design 2014. Here, the tibial plateau formed the xy-plane, while the z-axis runs vertically through the tibial knee center (Fig. 1).

As previously published, 14 defined landmarks were placed on the surface of the tibiae in order to analyze the 3D anatomy [21]. The anatomical axis was defined using the midpoints of the tibial shaft at the level of one and two thirds of the shaft length. The medial and lateral tibial slope could be identified by the two lines between the most proximal anterior and posterior points and the anatomical tibial axis [11]. The tibial torsion was defined by two lines (dorsal tangent to proximal tibia plateau and line between the deepest point of the incisura fibularis tibiae and the outermost point of medial malleolus) [23]. Numerous initial values including MPTA, medial and lateral sagittal slope, and tibial torsion were calculated using a Python script [21].

The virtual HTO was performed using biplanar cuts. The first cut in the coronal plane was made one centimeter behind the most ventral point of the tibial tuberosity, parallel to the anterior edge of the tibia. In the second step, the main osteotomy was performed from medial to lateral. The medial cut of the osteotomy was parallel to the medial slope and started distally by half of the maximum of the tibial plateau width (in average 35 mm). The target point of the osteotomy plane was 15 mm distal (z-axis) to the lateral tibial plateau. The hinge axis was defined at the lateral edge of the tibia and the cut ended 15 mm medially to the lateral cortex. The osteotomy gap was always opened by 8 mm (in the z-axis direction) at the most medial point to evaluate the printed cutting guides that should perfectly fit to the individual tibia. During 3D planning, only the MPTA was modified, while slope and torsion remained unchanged.

### Generation of 3D printed cutting guides/spacers and surgical realignment

**Fig. 2:**
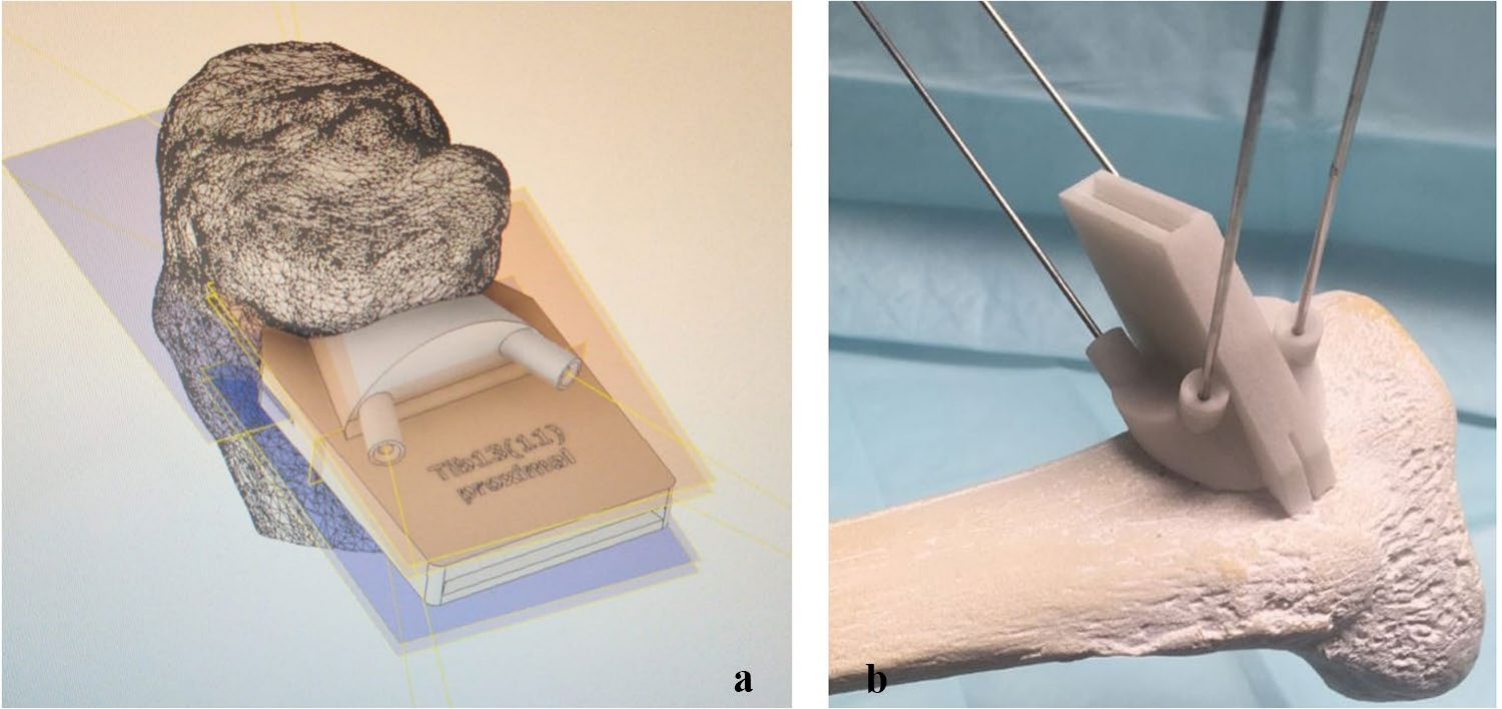
Patient-specific sawing guide designed in Inventor Professional 2020 (Autodesk Inc., San Rafael, CA, USA) for left tibia (**a**); Cutting block at curvature of the proximal right tibia (sawbone test; **b**)

The virtually osteotomized surface models were then converted to volume models using Catia V5 (Dassault Systèmes, Vélizy-Villacoublay, France) using a previously described method [22]. Based on these virtual planning models, PSIs and spacers were designed using the CAD-program Inventor Professional 2020 (Autodesk Inc., San Rafael, CA, USA; Fig. 2a).

**Fig. 3:**
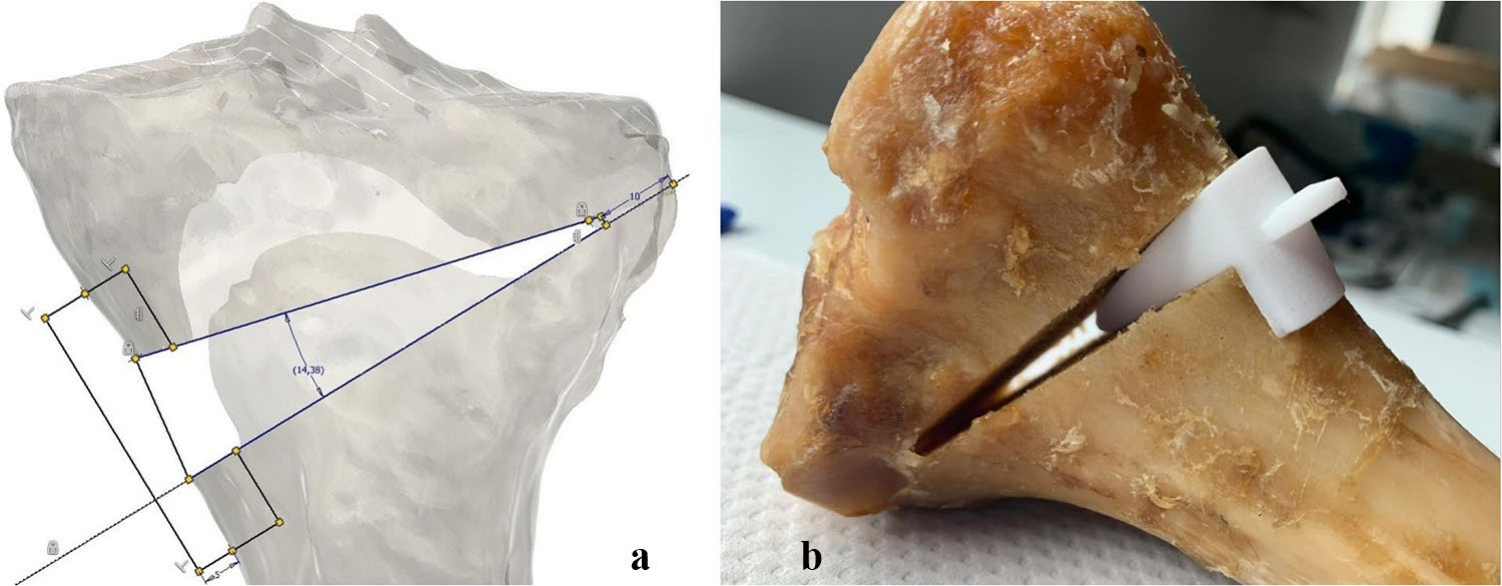
**a**: Design of a spacer in Inventor Professional 2020 (Autodesk Inc., San Rafael, CA, USA), **b**: Inserted spacer after osteotomy for a left tibia (dorsal view)

For each 3D model, the corresponding cutting block was specifically adapted to the curvature of the tibia. The cutting blocks were designed to be as small as possible to avoid the need for complete detachment of the medial collateral ligament (MCL) complex in real surgery. The sawing guides hence enabled the execution of the cut according to the predefined planning performed in Geomagic (see Figs. 2, 3).

During the actual sawing process, the template also prevented the saw blade from penetrating too deeply, so that the cut ended at 15 mm from the lateral tibial surface (Fig. 3). The guide itself was temporarily fixed to the tibia using four K-wires (2.5 mm).

A separate spacer/wedge was specifically designed for each bone based on the executed planning. The spacers allowed an optimal gap opening of 8 mm by inserting the wedge posteriorly to the plate. The curvature of the spacer allowed a defined positioning and easy removal after plate fixation.

The osteotomy was fixed with 3D printed reverse-engineered plates similar to TomoFix™ which, to simplify the verification of the resultant osteotomy, are used to prevent artifacts from appearing in CT scans. The plate was affixed via two proximal and distal screws. The wedge was then removed.

Using selective laser sintering (SLS) technology, the wedge, spacers, and osteotomy plates were additively manufactured using biocompatible polyamide. The medial open-wedge osteotomies were performed on a total of 13 tibial cadavers.

### Control of surgical realignment

After physical osteotomy of the tibiae with the specific instrumentation, the realigned specimen was scanned by CT once more (GE HD750 CT, General Electric, Boston, USA) with a slice thickness of 0.625 mm. The segmentation procedure was repeated on the DICOM data of the osteotomized tibiae in order to create the 13 tibial surface models (ImFusion Suite, ImFusion GmbH, Munich, Germany). The angles were determined again by setting the defined landmarks on the 3D bone models as described previously. The results were then compared with the original 3D plans. The deviations between plans and results were analyzed qualitatively using a distance-based false color analysis. The 3D plan and the surgical result were aligned using the pre-alignment function of the GOM Inspect Suite 2020 (GOM GmbH, Germany) in order to allow for surface comparison between the two. Here, the realigned anatomy is matched to the planned anatomy with the help of an automatic calculation of the best-fit. Because only the identical areas of the anatomies can be used for the regression, the proximal part of the repositioned tibia was separated using Inventor Professional 2020 (Autodesk Inc., San Rafael, CA, USA). The coordinate system was adapted and transferred to the entire anatomy. The surface of both models was then compared in the GOM Inspect Suite 2020 (GOM GmbH, Germany). The software computes the perpendicular distance of each polygonal point on the realigned anatomy to the planned anatomy. The software displays the deviation as a color plot. For quantitative evaluation, deviation in the realignment of more than 1.7 mm was defined as unacceptable. The ranges are displayed using the following color scheme: Very good (green) deviation up to 0.6 mm above/below, good (yellow/light blue) deviation of 0.6–1.2 mm above/below the planned position, acceptable range (orange/blue) 1.2–1.7 mm above/below, unacceptable range (red/dark blue) deviation of at least 1.7 mm above/below the planned position.

### Reliability of 3D HTO planning

In order to ensure high reliability, the same sequence of CT scan, segmentation and landmark setting according to preliminary studies was performed [21]. The 13 osteotomized tibiae models were aligned into the same predefined coordinate system and relevant angles of them were automatically calculated again via the same Python script.

Two investigators performed 3D HTO planning of the cadavers and performed the post-interventional measurement of the osteotomized tibiae using the scanned 3D models. As described, the aim of this surgical implementation was to achieve an HTO of 8 mm gap measurement and changing the MPTA while maintaining the medial and lateral slope and torsion. Accordingly, to the consistent 8 mm gap, the templates could evaluated better.

### Statistics

Each examiner placed the predefined landmarks on the cadaver tibia and performed an 8 mm osteotomy. Measured angles were given in absolute values. The same procedure was followed with the scanned HTO-tibiae. In both cases, differences across all 13 tibiae were reported as mean values (with standard deviations) between investigators [24]. The averaged values of the examiners were then compared with the planned and real HTO-values. The corresponding deviations were also indicated and further processed using Excel (Microsoft, Redmond, WA, USA).

## Results

### Reliability of the measurements

Regarding the reliability of the measurements, previous studies of the presenting group revealed an intraclass correlation (ICC) of > 0.75; except the tibial torsion (MPTA 0.98; medial slope 0.8; lateral slope 0.9; tibial torsion 0.69) [21]. The mean differences in two-observer measurements for the models in this study (3D planning and surgical outcomes) are displayed in Table 1.

**Table 1:** Mean differences between observers` measurements of 3D planning and surgical outcome

	MPTA	Medial tibial slope	Lateral tibial slope	Tibial torsion
Mean value	0.57	0.98	1.26	5.74
SD	0.50	0.61	1.21	4.6

*SD* standard deviation

### Deviation of 3D HTO planning versus surgical result

Compared to the 3D planning, the osteotomies showed an average MPTA deviation of 0.57° (SD ± 0.27). Medial and lateral slope differed by an average of 0.98° (SD ± 0.53) and 1.26° (SD ± 0.79), respectively, tibial torsion differed by an average of 5.74° (SD ± 3.24; Table 2). Overall, however, the measurements of both observers—apart from the tibial torsion—showed acceptable to good results.

**Table 2:** Difference and absolute mean difference between real und planned HTO in degrees (°)

Tibia No	MPTA	Medial tibial slope	Lateral tibial slope	Tibial torsion
1	0.25	1.42	3.03	6.18
2	0.46	0.45	0.89	6.81
3	0.83	0.97	0.95	3.12
4	0.28	1.97	0.02	5.64
5	0.94	0.4	1.39	4.62
6	0.85	0.79	0.61	5.66
7	0.14	1.19	1.44	12.67
8	0.85	0.79	0.61	4.89
9	0.41	0.98	2.21	1.16
10	0.31	0.37	0.63	11.63
11	0.84	1.69	1.88	2.84
12	0.78	1.39	1.52	5.52
13	0.63	0.35	1.27	3.91
Absolute mean value	0.57	0.98	1.26	5.74
SD	0.27	0.53	0.79	3.24

*SD* standard deviation

### False color analysis

**Fig. 4:**
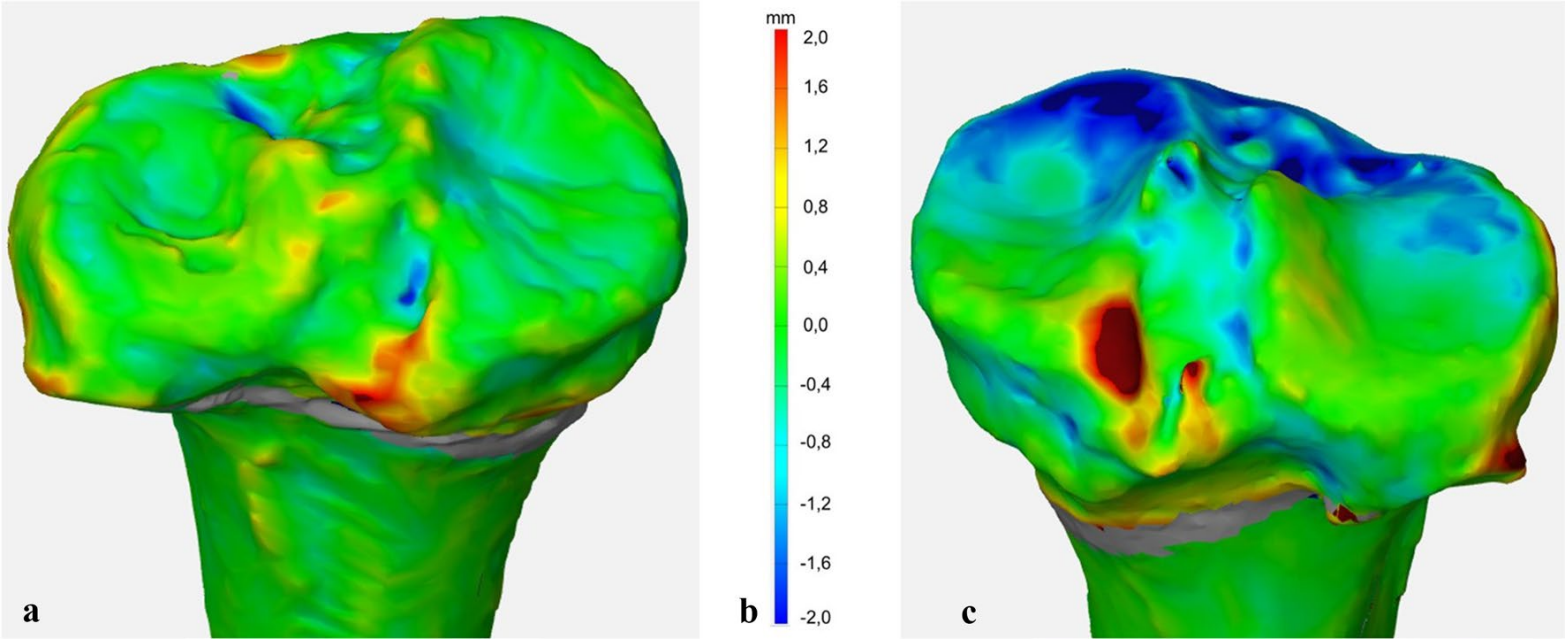
**a**: Very good results in surface comparison with minimal deviation between the plan and the surgical result. **b**: deviation in millimeters. **c**: ventral under-correction of surgical result compared to the planning

In the false color analysis using GOM Inspect Suite 2020 (GOM GmbH, Germany), a surface comparison was performed between the 3D plan and the surgical result. Seven tibiae had only minimal surface differences compared to the preoperative 3D plan which are most likely segmentation related color deviations (Fig. 4).

Six tibias were found to be under-corrected, three of them with respect to the MPTA and in two of them at the ventral tibial part (Table 3).

**Table 3:** False color analysis of surface deviations of the 13 tibiae and described deviations

Tibia	HTO
01	Very Good
02	Very good
03	Very good
04	Very good
05	General under-correction
06	Ventral under-correction
07	General under-correction
08	General under-correction
09	Good
10	Very good
11	Ventral under-correction
12	General under-correction
13	Very good

## Discussion

Overall, the results of the realignment surgery on cadaveric models using 3D plans and 3D printed cutting blocks are convincing. The 3D approach is superior when compared to conventional two-dimensional (2D) planning and implementation with accepted ranges of ± 3° MPTA [10]. Tibial knee joint angles (MPTA, medial and lateral slope) deviated in average by less than 1.3 degree from the preoperative planning in this study.

One of the main problems in 3D planning and implementation of osteotomies is that the ideal technique of 3D anatomy analysis, the ideal target and the accepted range of accuracy are still subject of discussion in current literature. Even in the 2D there are planning methods which use the Mikulicz line in relation to the tibial plateau or using the Hip-Knee-Angle [25–28]. However, the strong results in clinical studies showing a deviation of only 0.1° between planning and surgical outcome when using 3D PSI in high tibial osteotomy could not be replicated in this study [29]. In addition, a meta-analysis demonstrates no significant improvement in accuracy by PSI compared with the conventional technique even though outliers were reduced by the presented technique [25].

Results in tibial torsion, a relevant value representing the third dimension, are not included in most published studies investigating PSI in osteotomy [15, 16, 29, 30]. One reason for deviations of tibial torsion in this study (up to 12.67°) could be the manual selection of landmarks on the 3D bone surface model. Liodakis et al. demonstrated that torsion control in the case of fracture or osteotomy is prone to error using 2D imaging only. 3D imaging is listed here as a possible solution [31, 32]. Goleski et al. also described poor torsional reliability of navigated lower limb alignment in HTO, while Liodakis et al. compared different methods of measuring torsion on 2D CT slice images; good values were shown across all measurements [23, 33]. However, our group`s previous work on 3D analysis of lower limb alignment showed that manual landmark determination of tibial torsion led to low reliability [21]. In conclusion, a more precise 3D landmark definition seems to be necessary for tibial torsion. Ideally, this will be achieved by automatic landmark recognition, a method which was recently published by Stephen et al. [34].

False color analysis of surface deviations and angle measurements revealed unintended slope changes in some specimens. Under-correction of the anterior tibia (two cases) is a possible consequence of wedges inserted posteriorly only. Under-correction of MPTA occurred in four specimen which could be explained by post-sintering after removal of the wedge as described by Valkering et al. [35]. Fresh, frozen cadaver bone is more rigid and therefore harder to deform, so these problems are likely to be less severe in vivo. Both aspects could be addressed by bigger allograft wedges or 3D printed scaffolds in future studies.

One limitation of this study is that the osteotomy was performed on cadaveric tibiae only. This means that there was no fibula or soft tissue. Accordingly, the lateral malleolus landmark was placed in the deepest point of the Incisura fibularis tibiae and considering the multi-observer measurements, this landmark appears to have a low reliability, resulting in low accuracy in the measurement of tibial torsion.

Specimens without soft tissue were selected to facilitate the segmentation process and to reduce differences due to repeated CT scans and in the segmentation process. With a slice thickness of 0.65 mm and thresholding segmentation, the differences in the creation of the 3D model were estimated to be minimal [22, 36]. Conclusions about possible soft tissue forces are therefore not possible [37, 38]. At the same time, the absence of soft tissues enabled perfect fitting of the template to the bone.

However, in accordance with previous studies, results of angle measurements and false color analysis indicate a high accuracy for the evaluated 8 mm-osteotomies performed with 3D printed cutting blocks [15–17]. Jud et al. showed that a slight deviation of the template does not seem to have a relevant effect on coronal alignment in HTO [39]. Templates can be designed and used according to desired target angles [40]. Even the problem of posterolateral hinge positions in HTO increasing the tibial slope was avoided in this study by using 3D printed PSIs [41, 42].

In future studies, templates adapted to the 3D model will allow different heights of osteotomy gaps and with oblique osteotomy planes they will allow multidimensional changes. Variously shaped wedges also offer a wide range of correction possibilities for open-wedge osteotomies.

First in vivo results also showed similar advantages in accuracy with PSI for femoral osteotomies [43]. In addition to improving accuracy, 3D printed PSIs could reduce intraoperative fluoroscopy time and surgical time [44, 45].

An important point of this study is the execution of the essential steps in the planning and analysis process by orthopedic surgeons. An uninfluenced analysis of these new promising possibilities of PSI, independent of the manufacturing companies, is necessary for a critical scientific discussion.

## Conclusion

With 3D printed PSIs and spacers, 3D planning of HTO can be realized with high accuracy. Only minor deviations were recorded in the surgical procedures using relatively small 3D printed PSIs. Some deviations in the 3D angle measurement are due to the manual nature of these measurements and can soon be improved by automation. False color analysis can reveal deviations from the 3D plans and helps to improve the procedure. Based on the present results, 3D cutting templates appear to be a promising tool for optimizing and simplifying HTOs, but further studies on 3D printed wedges and the required size of the cutting blocks are warranted.
